# Co-Exposure with the Herbicide 2,4-D Does Not Exacerbate *Batrachochytrium salamandrivorans* Infection in the Italian Crested Newt (*Triturus carnifex*)

**DOI:** 10.3390/ani15121777

**Published:** 2025-06-17

**Authors:** Eduardo Fernández Meléndez, Léa Fieschi-Méric, Elin Verbrugghe, Ellen Blomme, Michael Fahrbach, Manuel E. Ortiz-Santaliestra, Frank Pasmans, An Martel

**Affiliations:** 1 Wildlife Health Ghent, Department of Pathobiology, Pharmacology and Zoological Medicine, Faculty of Veterinary Medicine, Ghent University, 9820 Merelbeke, Belgium; elin.verbrugghe@ugent.be (E.V.); ellen.blomme@ugent.be (E.B.); frank.pasmans@ugent.be (F.P.); an.martel@ugent.be (A.M.); 2 Niedernhaller Str. 8/2, 74653 Criesbach, Germany; michael.fahrbach@gmx.de; 3 Instituto de Investigación en Recursos Cinegéticos (IREC), CSIC-UCLM-JCCM, Ronda de Toledo 12, 13005 Ciudad Real, Spain; manuele.ortiz@uclm.es

**Keywords:** agrochemicals, amphibian conservation, emerging infectious diseases, pollution, chytridiomycosis, newts

## Abstract

Amphibians face a multitude of threats and therefore make a prime example of the current biodiversity crisis. Multiple amphibian stressors in anthropogenic landscapes include infectious diseases and agrochemicals. Synergic effects between these stressors may increase the negative impact of infections on amphibian health. In a 56-day trial, we assessed the impact of co-exposure to the herbicide 2,4-dichlorophenoxyacetic acid (2,4-D) and the pathogenic fungus *Batrachochytrium salamandrivorans* (*Bsal*) on infection parameters (infection intensity and disease severity) and health (body condition and telomere length) in Italian crested newts (*Triturus carnifex*). Twenty days post-inoculation with *Bsal*, newts were either exposed to 2,4-D for 12 days or not exposed (control). Most newts developed high infection loads that steadily increased towards the end of the trial. While body condition was negatively correlated with pathogen burden, only one out of 23 animals died. Telomere length remained unaffected by the pesticide and the pathogen. The 2,4-D treatment did not exacerbate *Bsal* infection. Most newts survived almost two months with significant pathogen loads; thus, even in a pesticide-infested environment, *T. carnifex* could be an important long-term *Bsal* reservoir for co-occurring species on the Italian peninsula, a urodele diversity hotspot.

## Simple Summary

Amphibians are among the most threatened animals on the planet, facing dangers from pollution, habitat loss and infectious diseases. In this study, we focused on the Italian crested newt, a type of salamander, to see if exposure to a commonly used weed killer called 2,4-D would make them more vulnerable to a deadly skin disease caused by a fungus. We exposed newts to both the herbicide and the fungus and monitored their health over almost two months. We found that while many newts became infected and their health was affected by the fungus, the herbicide did not make the disease worse. Most newts survived the trial, even with high levels of infection. This suggests that Italian crested newts can tolerate short-term pollution from this herbicide, but they might still carry and spread the disease to other, more sensitive amphibians. Our findings help us understand how pollution and disease interact in nature, and this knowledge can guide efforts to protect amphibians and maintain healthy ecosystems.

## 1. Introduction

Amphibians are the most endangered and rapidly declining vertebrate group on Earth, with 41% of species threatened with extinction [1,2]. The main cause of these declines is habitat loss and degradation, notably through agriculture (77% of species impacted), but climate change, invasive species, pollution and diseases caused by a wide range of parasites (29%) also constitute important threats [3,4,5,6,7]. Recent research suggested that some of these threats may interact synergistically, thus multiplying their impacts on amphibians [8,9]. As such, pesticides and infectious diseases may have stronger impacts on amphibians when combined, but their effects on hosts are poorly understood and deserve further investigation [10,11].

The skin disease chytridiomycosis is leading to global declines in amphibians [12]. Chytridiomycosis is caused by the fungi *Batrachochytrium dendrobatidis* (*Bd*) and *Batrachochytrium salamandrivorans* (*Bsal*) [13,14]. These fungi can be transmitted through multiple pathways, by direct contact with or necrophagy of infected individuals, or by exposure through the environment [15,16]. *Bsal* disrupts the epidermis through ulceration, leading to death in susceptible urodeles [14,17]. *Bsal* has been reported only in Eastern Asia, where it presumably originated from, and in Northwestern Europe, where it may have been introduced through the pet trade [18,19]. Since its first detection in 2013 in the Netherlands, *Bsal* has caused massive declines in urodele species in Belgium, France, Germany, and Spain [14,20,21,22]. *Bsal* currently appears to be absent from the Italian peninsula, a urodele diversity hotspot in Europe [23]. Previous research showed variable outcomes of pesticide exposure on amphibians in response to chytrid infections caused by *Bd,* ranging from disease exacerbation [10,24] to no obvious impact [25] or even reduced fungal proliferation and altered infection dynamics [26].

Pesticide exposure constitutes another important driver of amphibian declines [27]. In Europe, over 300,000 tons of pesticides were used in 2022, with France, Spain, Germany, and Italy recording the highest volumes [28]. One of the most widely used pesticides worldwide is the herbicide 2,4-Dichlorophenoxyacetic acid (2,4-D), with about 150,000 tons globally spread across fields every year [29]. Its use in Europe increased by 40% over the first decade of the 21st century [30]. Although numerous studies demonstrated the toxicity of 2,4-D in amphibians [31,32], this pesticide remains widely used in areas where amphibians are present [30]. Described toxicity effects in amphibians include behavioral alterations, changes in growth patterns [33] and reduced survival at metamorphosis [34]. In humans, exposure to 2,4-D is associated with telomere length reduction, a process linked to premature aging and increased disease vulnerability [35]. Telomere length is reduced with each cellular division in a normal process towards cell senescence; however, telomere attrition can also happen because of different stress sources, including pesticide exposure as observed in fish [36] or humans [37]. However, the effects of pesticides on amphibian telomeres remain uninvestigated.

Herbicide 2,4-D has been shown to affect the immune response, reducing, for instance, lymphocyte stimulation in mice [38] or phagocytic activity in earthworms [39]. Despite no immunotoxic effects of 2,4-D having been studied on amphibians, we hypothesize that exposure to the herbicide could contribute to increasing the susceptibility of amphibian hosts to *Bsal*. With this purpose, we explored the impact of exposure to 2,4-D on the course of infection of *Bsal* in a supposedly susceptible newt inhabiting the Italian peninsula. We used the Italian crested newt (*Triturus carnifex*) as a model species, given its relatedness to known *Bsal* susceptible species [14]. This semi-aquatic salamander is native to the Italian and Western Balkan peninsulas, Austria and the Czech Republic [40] and is becoming invasive in other parts of the European continent, such as Switzerland [41] and the Netherlands [42,43]. This new invasive range overlaps with the native distribution of its congener *T. cristatus*, which is susceptible to *Bsal* [44] and currently decreasing [2]. In this study, we assess the impact of 2,4-D exposure on *Bsal* infection intensity, disease severity and overall animal health.

## 2. Materials and Methods

### 2.1. Experimental Setup

An overview of the experimental setup is presented in Supplementary Figure S1. In total, 24 subadult, captive-bred Italian crested newts (*Triturus carnifex*; 17 females, 28 males) were randomly divided into 4 treatment groups: **2,4-D + *Bsal***: exposed to *Bsal* and 2,4-D (*n* = 12); **2,4-D**: only exposed to 2,4-D (*n* = 11); ***Bsal***: only exposed to *Bsal* (*n* = 11); and **Neg**: sham-treated negative controls (*n* = 11). All animals were weighed on a microscale (WT-CS200 Digital Pocket Scale, WeighTAJ, Zhejiang, China) and photographed ventrally and dorsally before the start of the experiment. Animals were placed individually in tissue-lined terraria at 15 °C, optimal for *Bsal* growth and pathogenicity. Each terrarium contained a plastic pipe to provide shelter. The animals were fed calcium- (Repti-Calcium with D3, Zoo Med, St. Louis, CA, USA) and vitamin- (Reptivite Vitamin D3, Zoo Med) supplemented crickets (*Acheta domestica*) and buffalo worms (*Alphitobius diaperinus*) ad libitum.

On Day 0, 2,4-D + *Bsal* and *Bsal* groups were exposed to *Bsal* (AMFP13/01) by bathing the newts in 1 mL of 8.8 × 10^3^ *Bsal* spores for 24 h at 15 °C to provide a low infection dose [14]. Neg and 2,4-D groups were sham-treated. After exposure, all animals were sampled weekly by collecting a skin swab (CLASSIQSwabs 160C, COPAN, Murrieta, CA, USA) to quantify *Bsal* load. From Day 20 until Day 32, animals from groups 2,4-D + *Bsal* and 2,4-D were exposed to 2,4-D by continuously bathing in a 100 mL solution containing 0.3 mg/L 2,4-D. This pesticide level is consistent with the peak Predicted Environmental Concentration of 2,4-D in surface waters following pesticide applications, according to models used by the substance registration in the pesticide EU market (0.24 mg/L) [45]. Therefore, we used an environmentally relevant concentration which, in principle, would not be expected to affect animals’ survival as it is approximately 27 times lower than the lowest median lethal concentration for the substance on amphibians (8.05 mg/L for *Duttaphrynus melanostictus* larvae) [46]. The 2,4-D solution was renewed daily in order to maintain a constant concentration of pesticide throughout the duration of exposure. Animals from the *Bsal* group and the negative control group were sham-bathed in daily renewed water to ensure comparable conditions. Feeding was withheld during this window of exposure to limit waste accumulation.

At Day 56, eight weeks after *Bsal* exposure, the experiment ended. All animals were photographed, weighed and euthanized through an intracoelomic injection of 20% sodium pentobarbital (KELA; except for one individual that died on Day 47). Before euthanasia, a mouth swab to determine telomere length was collected. Swabs were stored frozen until further analysis. Tissues were collected and preserved for use in future complementary studies, in accordance with the principles of reduction and refinement.

### 2.2. Morphological Metrics

Ventral and dorsal photographs of the animals on millimeter paper were used to measure individual snout–vent length (SVL) using the software ImageJ version 1.54d. The average of three repeated SVL measures was used in further analyses. Body condition was estimated using the body mass index (BMI) measured as mass (g)/SVL (mm)^2^ [47,48]. Changes in body condition over time were estimated as the difference between final and initial BMI values (delta BMI, Day 56 − Day 0).

### 2.3. Bsal Load, Infection Intensity and Disease Severity Quantification

**Figure 1:**
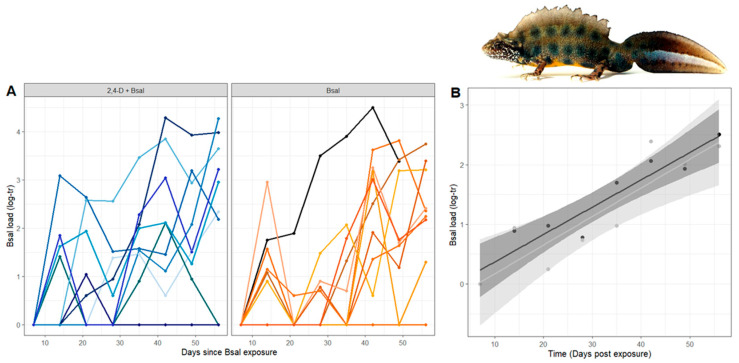
Individual *Bsal* loads curves separated by treatment (2,4-D + *Bsal,* blue vs. *Bsal,* orange) and highlighting the individual that died (TC9, in black on the right panel) (**A**). Average *Bsal* load per treatment over time (2,4-D + *Bsal* treatment in black, and *Bsal* treatment in light gray) (**B**). The shaded area around each line represents the 95% confidence interval (CI) for the mean *Bsal* load at each time point. *Bsal* load is log10-transformed (log-tr).

Fungal DNA was extracted from the swabs using a PrepMan^®^ Ultra Sample Preparation reagent (Applied Biosystems, Ghent, Belgium). The number of *Bsal* genomic equivalents was estimated using quantitative real-time PCR (qPCR), following the protocol from Blooi et al. [49]. The total *Bsal* load (pathogen burden) was measured as the area under the curve of the log10-transformed *Bsal* load (log10 (GE)) drawn from weekly qPCRs (Figure 1A).

Some authors suggest that the sole use of qPCR data may be a misleading indicator of chytridiomycosis disease status [50,51], so we supplemented our pathogen burden metric (total *Bsal* load) with two previously described indexes that distinguish between the early response to infection and the subsequent full ramifications of the disease: *infection intensity* [52], defined as the individual-specific slope to peak *Bsal* load within the first four weeks post-exposure, reflecting the early dynamics of pathogen proliferation, and *disease severity,* defined as a composite index incorporating four standardized components across the population: (i) total *Bsal* load (area under the qPCR curve), (ii) latency to peak infection (days to maximum load), (iii) change in body mass index (ΔBMI), and (iv) survival indicator (time of death relative to experiment duration). Post-mortem *Bsal* loads were replaced by the last recorded value to calculate pathogen burden. The *disease severity* index was adapted to include a survival indicator (time between the end of the experiment and the day of natural death, divided by the duration of the experiment; Supplementary Materials and Methods).

### 2.4. Telomere Length Assay

Being a proxy for exposure to stress, telomere length was assessed as a health indicator. Genomic DNA was extracted from buccal swabs using the DNeasy Blood and Tissue kit (Qiagen, Hilden, Germany), following the manufacturer’s protocol. DNA quantity and purity were assessed using a Nanodrop 1000 spectrophotometer (ISOGEN, Wilmington, DE, USA), and a qPCR was used for measuring relative telomere length [53]. Relative telomere length was calculated as the ratio (T/S) of telomere repeat copy number (T) to ultra-conserved element UCE359 copy number [54], normalized to a DNA mix reference sample.

Reactions were carried out in 15 µL volumes containing 5 µL DNA (varying concentrations of DNA for serial dilutions of the standard curve and 1/1000 dilutions for unknown samples), with two sets of primers: TelF/TelR to amplify the telomere region and UCE359F/UCE359R as a copy number reference control. For telomere qPCR, each reaction contained 7.5 µL of SensiMix^TM^ SYBR^®^ (Bioline, Essex, UK), 300 nM TelF primer (5′-GGT TTT TGA GGG TGA GGG TGA GGG TGA GGG TGA GGG T-3′), 400 nM TelR primer (5′-TCC CGA CTA TCC CTA TCC CTT CCC TAT CCC TAT CCC TA-3′), BSA (250 ng/µL) and HPLC water, resulting in a total reaction volume of 15 µL. A touch-down approach was used, including a 95 °C step for 10 min, followed by 6 cycles of denaturation at 95 °C for 15 s, annealing for 1 min with the temperature decreasing from 62 °C to 59.5 °C by 0.5 °C per cycle, and extension at 72 °C for 30 s. Subsequently, 34 amplification cycles were performed, each consisting of denaturation at 95 °C for 15 s and annealing/extension at 58.3 °C for 1 min with signal acquisition at the end of the 58.3 °C step. For UCE359 qPCR, the reaction mixture included 7.5 µL of SsoAdvanced Universal SYBR Green Supermix (Bio-Rad, Hercules, CA, USA), 500 nM UCE359F primer (5′-ATC TGA GAC TTG TGA CAT-3′) and 500 nM UCE359R primer (5′-GTG TTA ATT GGT AAT GAC TAT T-3′) and HPLC water, resulting in a total reaction volume of 15 µL. A two-step thermal cycling protocol was followed, including 95 °C for 5 min, followed by 40 cycles of 95 °C for 30 s and 55 °C for 30 s, with signal acquisition at the end of the 55 °C step. Melt curves for both qPCRs were generated by increasing temperatures from 65 °C to 95 °C in 0.5 °C steps.

Reaction efficiencies were assessed by including a standard curve on every plate, created through six 1:2 serial dilutions of a DNA mix (starting at 12.5 ng). A 1/1000 dilution of the DNA mix served as the reference sample on each plate. This reference sample was used to calculate the relative telomere length. All samples, including the standard curve, were run in triplicate, and average values were used to calculate the T/S ratios. Triplicate variability was evaluated, and replicates deviating by more than 0.5 Cq from the mean were excluded. After correction for inter-plate variation, the following formula was used to measure the relative telomere length: 2^−ΔΔCt^, where ΔΔCt = (Ct telomere − Ct UCE_359_)_sample_ − (Ct telomere − Ct UCE_359_)_reference_.

### 2.5. Statistical Analysis

Statistical analyses were performed using R version 4.3.2 (R Core Team, 2023); all data and code are publicly available in the Figshare repository: https://figshare.com/s/34c5dcc160a83df58ece (accessed on 8 June 2025). Statistical tests were deemed significant if associated with *p*-values below a 0.05 threshold.

First, we checked whether *Bsal* susceptibility differed between male and female newts using t-tests. To investigate whether exposure to 2,4-D influenced the response to *Bsal,* linear models were built with *infection intensity* and *disease severity* as the response variables, and with the initial BMI (Day 0), pesticide exposure (2,4-D), and their interaction as explanatory variables. The effect of the covariates included in these models was tested with an analysis of covariance (ANCOVA) because their residuals were normally distributed and homoscedastic.

To determine the impact of *Bsal* and 2,4-D (co-)exposure on health (body condition and telomere length), linear models were built with delta BMI (Day 56 − Day 0) and post-exposure (Day 56) telomere length as response variables, and treatment group (Neg; *Bsal*; 2,4-D; 2,4-D + *Bsal*) as the explanatory variable. The effect of treatment group on the average and within-group variation in each response variable was tested through analyses of variance (ANOVA) and Barlett tests, followed by post hoc F-tests, respectively.

Correlation tests were used to determine whether body condition or telomere length following *Bsal* exposure were correlated with *Bsal* total load, *infection intensity* or *disease severity*; if the assumption of normality associated with Pearson’s correlation was violated, a non-parametric Kendall correlation test was used. Note that the correlation between body condition and disease severity was not investigated since our custom-built disease severity index included body condition change.

## 3. Results

### 3.1. Susceptibility to Bsal Infection Varies Between Individual Italian Crested Newts

Weekly quantification of the *Bsal* load by qPCR demonstrated important variation in *Bsal* susceptibility between individuals: 19/23 newts (9 in the 2,4-D + *Bsal* treatment and 10 in the *Bsal* treatment) became infected after exposure, but only one animal died during the trial (at 7 weeks post-inoculation). This animal developed a high infection load of 31,550 genomic equivalents (GEs) and showed clear signs of skin ulceration and epidermal erosion following exposure. Individual infection trajectories were highly variable between individuals (Figure 1A) but, on average, increased steadily over the course of the experiment, and the majority of animals developed high infection loads by the end of the trial (1044 GE at Day 56; Figure 1B). *Bsal* susceptibility was not different between males and females (*t*-tests; *Bsal* load, *t* = 1.21, *df* = 14.82, *p*-value = 0.246; infection intensity, *t* = 0.97, *df* = 4.46, *p*-value = 0.382; disease severity, *t* = 0.84, *df* = 11.63, *p*-value = 0.420).

### 3.2. Bsal Load, Intensity of Infection and Severity of Chytridiomycosis Are Not Affected by Short-Term 2,4-D Exposure

**Figure 2:**
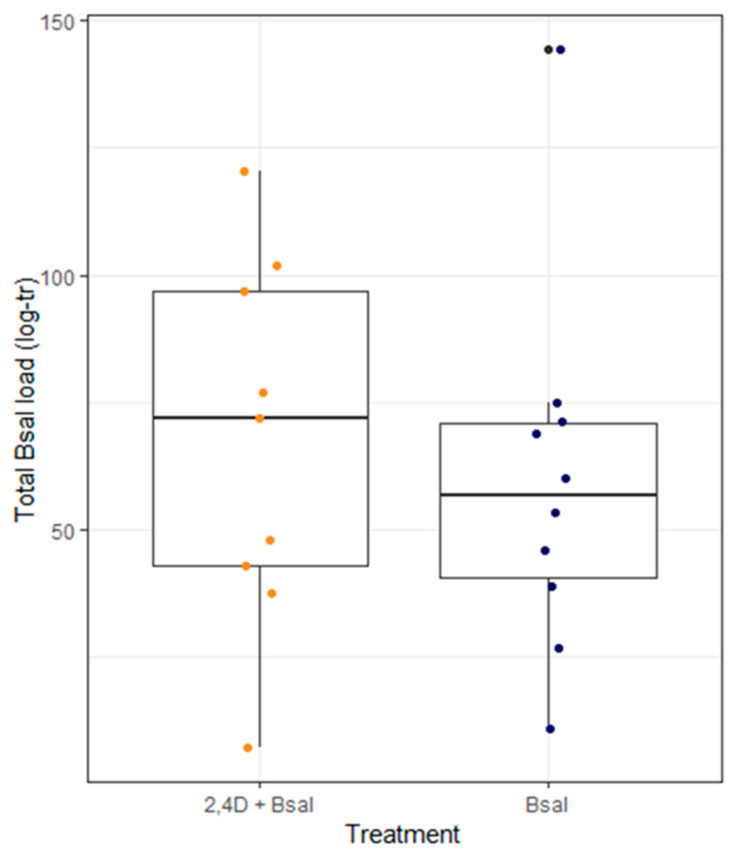
Total *Bsal* load per 2,4-D treatment group (2,4-D + *Bsal* vs. *Bsal*). Each dot represents an individual. The black line is the median, the box shows the interquartile range (IQR), and the whiskers indicate the range of the data excluding outliers. Total *Bsal* load was measured as the area under the curve of *Bsal* load drawn from weekly qPCRs. Three individuals in the 2,4-D + *Bsal* treatment and one individual in the *Bsal* treatment did not become infected with *Bsal* and are thus not represented here. *Bsal* load is log10-transformed (log-tr).

Among *Bsal*-exposed newts, co-exposure to 2,4-D and initial body condition did not significantly affect *Bsal* total load (ANCOVA; 2,4-D, *F*_(1,15)_ = −0.94, *p*-value = 0.363; BMI, *F*_(1,15)_ = −1.46, *p*-value = 0.166; interaction term, *F*_(1,15)_ = 0.91, *p*-value = 0.377; Figure 2), infection intensity (ANCOVA; 2,4-D, *F*_(1,15)_ = −1.19, *p*-value = 0.253; BMI, *F*_(1,15)_ = −1.88, *p*-value = 0.079; interaction term, *F*_(1,15)_ = 1.15, *p*-value = 0.270) or *Bsal* disease severity (ANCOVA; 2,4-D, *F*_(1,15)_ = −0.61, *p*-value = 0.548; BMI, *F*_(1,15)_ = −0.64, *p*-value = 0.533; interaction term, *F*_(1,15)_ = 0.59, *p*-value = 0.562; Supplementary Figure S2).

### 3.3. Newt Health Is Determined by the Total Bsal Load but Not by Exposure to 2,4-D

**Figure 3:**
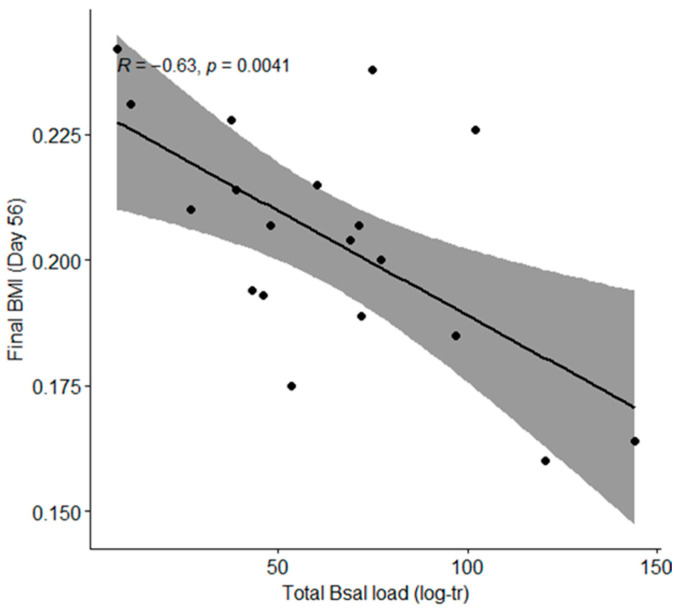
Correlation between body condition at the end of the experiment (Day 56) and total *Bsal* load among *Bsal*-exposed individuals. Each dot represents an individual. The black line shows the linear regression, and the shaded area is the 95% confidence interval. Total *Bsal* load was measured as the area under the curve of *Bsal* load drawn from weekly qPCRs. *Bsal* load is log10-transformed (log-tr).

We used body condition change (delta BMI) over time and telomere length as proxies for individual health. Body condition at the end of the experiment was negatively correlated with *Bsal* total load over the trial period (Pearson correlation; *t* = −3.32, *df* = 17, *p*-value = 0.004; Figure 3) but not correlated with infection intensity (Pearson correlation; *t* = 0.70, *df* = 17, *p*-value = 0.496; Supplementary Figure S3A). Average body condition variation over time was not affected by exposure to *Bsal* and/or 2,4-D (ANOVA; *F*_(3,41)_ = 2.10, *p*-value = 0.115). The variance in body condition change over time was not significantly different among groups (Barlett test; *K*^2^ = 7.33, *df* = 3, *p*-value = 0.062; Supplementary Figure S4A).

Relative telomere length was not affected either by exposure to *Bsal* and/or 2,4-D (ANOVA; *F*_(3,40)_ = 0.77, *p*-value = 0.517). The variance in telomere length was not significantly different among groups (Barlett test; *K*^2^ = 3.25, *df* = 3, *p*-value = 0.355; Supplementary Figure S4B). Telomere length at the end of the experiment was not correlated with *Bsal* total load (Pearson correlation; *t* = −0.36, *df* = 16, *p*-value = 0.721), *Bsal* infection intensity (Pearson correlation; *t* = −0.05, *df* = 16, *p*-value = 0.961) nor disease severity (Kendall correlation; *z* = 0.303; *p*-value = 0.762; Supplementary Figure S3B–D).

## 4. Discussion

This study contributes to our understanding of the short-term consequences of emerging infectious diseases in a polluted world. Our results show a range of variable *Bsal* susceptibility in *T. carnifex*, with exposure to *Bsal* causing no infection in some newts, high infection loads in others and even being lethal for one individual. Body condition at 56 days after inoculation was significantly correlated with pathogen burden across the duration of the trial, indicating a marked subclinical cost of infection. This correlation aligns with principles of ecological energetics, where pathogen challenge imposes metabolic costs that divert energy from somatic maintenance and growth. This subclinical cost of infection may reflect a resource allocation trade-off, whereby hosts balance energy investment in immune defenses against vital physiological processes [55]. In amphibians, chytrid infections are known to disrupt osmoregulation and increase cutaneous ion loss [56], necessitating compensatory energy expenditure for electrolyte balance and tissue repair [12,13]. Consequently, heavily infected or smaller individuals may be particularly affected, as their energy reserves are depleted more rapidly [55,57].

Telomere length was neither affected by the exposure to *Bsal* nor to 2,4-D, but this may be due to the relatively short term of the trial, with telomere attrition often being considered a longer-term cost of infection. Consequently, the implications of this phenomenon in terms of telomere-shortening-related aging within natural animal populations are likely to be strongly underestimated [55]. This variation suggests complex and diverse host–pathogen interactions, which should be investigated further to understand how host factors influence *Bsal* infection risk. Since the experiment was terminated at 56 days, we cannot exclude that over a longer term, a substantial proportion of the infected animals would have died or shown telomere attrition. Indeed, infection loads were steadily increasing towards the end of the trial, and body condition at the end of the trial was negatively correlated with *Bsal* load. This pattern highlights the importance of longer-term monitoring, as studies in other urodeles have shown that *Bsal* infection can persist for months with delayed mortality and subclinical effects. Previous cases with European species like *Ichthyosaura alpestris* show that they can maintain high *Bsal* loads for extended periods, acting as reservoirs and facilitating ongoing transmission, as opposed to the rapid mortality in hypersusceptible species [20,58]. Environmental persistence of *Bsal,* including infectious zoospores and carcasses, further supports the need for long-term studies to capture delayed population impacts and chronic health costs [16,55]. Thus, while our 56-day trial provides valuable short-term insights, only extended monitoring can reveal the full scope of *Bsal’s* impact on newt populations. Regardless of individual newts’ fate, prolonged survival of heavily infected newts in the amphibian community poses a critical challenge in disease management, as demonstrated by Canessa et al. [59]. Their models assessing the impact of *Bsal* reservoirs revealed the complex dynamics of pathogen persistence and spread.

This study did not demonstrate any negative effect of exposure to 2,4-D on Italian crested newt body condition and telomere length, be it through the herbicide action alone or through combined effects with *Bsal* infection. Several studies have investigated the toxicity of 2,4-D on amphibians, the majority of which point to either sublethal or even lethal effects of the herbicide on these animals [60]. However, those studies usually measure pre-metamorphic stages, whose sensitivity to chemicals has been observed to be generally higher than that of post-metamorphic stages [61]. For the specific case of 2,4-D, these age-dependent differences in sensitivity have been noted even at different developmental points within the embryonic phase in *Xenopus laevis* [62]. Van Meter et al. [63] reported changes in the hepatic metabolome profile in green frog (*Lithobates clamitans*) juveniles, but the exposure scenario used in that study (exposure via contact with previously treated soils) is not comparable to our study. Using aquatic exposures like in the present study, Zaffaroni et al. [64] reported no mortality of *T. carnifex* adults exposed during 90 days at 25mg/L 2,4-D (i.e., 83 times higher than the concentration used in the present study). Lajmanovich et al. [65] reported that South American toad (*Rhinella arenarum*) adult males immersed for 48 h (i.e., one sixth of the exposure time in the present study) in a solution with 20 mg/L 2,4-D (i.e., 67 times higher than what we used) showed a significant increase in the activity of the metabolic enzyme glutathione-S-transferase and DNA damage, and a reduced heterophil-to-lymphocyte ratio, which could be indicative of a stress response. In general, the absence of observable effects of 2,4-D on subadult Italian crested newts is consistent with what was expected from the fact that the exposure level was environmentally relevant.

## 5. Conclusions

We found Italian crested newts to be susceptible to *Bsal* infection, with high-level infections reducing the newts’ body condition and limited mortality observed up to 56 days post-pathogen exposure. Apart from potential population-level implications for the species itself, infected Italian crested newts could thus play a role in maintaining and amplifying infections in amphibian communities in the urodele hotspot of Italy and their invasive range. Exposure to the herbicide 2,4-D had no obvious impact on *Bsal* infection and newt health, suggesting tolerance to short-term herbicide pollution in its breeding ponds.

## Data Availability

The data that support the findings of this study are available as described in the “Supplementary Materials” section or upon request from the corresponding author.

## Supplementary Materials

The following supporting information can be downloaded at https://www.mdpi.com/article/10.3390/ani15121777/s1, **Supplementary Figure S1:** Experimental set-up. Individuals were initially (Day 0) exposed to *Bsal* or maintained as negative controls; after 20 days, half of each group was exposed to 2,4-D (Day 20). Skin swabs were collected weekly to quantify *Bsal* load through qPCR. The trial ended 8 weeks after initial exposure to *Bsal* (Day 56). Morphometric measures were recorded at Day 0 and Day 56; buccal swabs to measure telomere length through qPCR were recorded on Day 56; **Supplementary Figure S2.** Relation between initial body condition (Day 0) and total *Bsal* load (A), infection intensity (B) and disease severity (C) following exposure to *Bsal* (where each dot represents an individual, the black line shows the linear regression and the shaded area is the 95% confidence interval). Relation between 2,4-D treatment (2,4-D + *Bsal* vs. 2,4-D) and infection intensity (D) and disease severity (E) following exposure to *Bsal* (where each dot represents an individual, the black line is the median, the box shows the interquartile range (IQR), and the whiskers indicate the range of the data excluding outliers). The relation between 2,4-D treatment and total *Bsal* load is presented in Figure 2; **Supplementary Figure S3.** Relation between final body condition and infection intensity (A). Relation between final telomere length and total *Bsal* load (B), infection intensity (C), and disease severity (D). Each dot represents an individual. The black line shows the linear regression and the shaded area is the 95% confidence interval; **Supplementary Figure S4.** Body condition change (delta BMI over the whole experiment) (A) and between variation in relative telomere length at the end of the experiment (B) across treatment groups. Each dot represents an individual. The black line is the median, the box shows the interquartile range (IQR), and the whiskers indicate the range of the data excluding outliers. The R code to build disease quantification indexes and experiment raw data table is available in the Figshare repository: https://figshare.com/s/34c5dcc160a83df58ece (URL accessed on 8 June 2025).

## Abbreviations

The following abbreviations are used in this manuscript:

*Bsal*	*Batrachochytrium salamandrivorans*
*Bd*	*Batrachochytrium dendrobatidis*
2,4-D	2,4-dichlorophenoxyacetic Acid
BMI	Body Mass Index
qPCR	Quantitative Polymerase Chain Reaction
GE	Genomic Equivalent
SVL	Snout–Vent Length
UCE	Ultra-Conserved Element
ANOVA	Analysis of Variance
ANCOVA	Analysis of Covariance
DNA	Deoxyribonucleic Acid
T/S	Telomere/Single-copy Gene Ratio
