# Supplementary material for: Co-Exposure with the Herbicide 2,4-D Does Not Exacerbate Batrachochytrium salamandrivorans Infection in the Italian Crested Newt (Triturus carnifex)
Source: Animals (Basel). 2025 Jun 17;15(12):1777. doi: 10.3390/ani15121777 (PMC12189190; doi:10.3390/ani15121777)
Supplement: Supplementary file 1 [file animals-15-01777-s001.zip › animals-3642303-supplementary.docx]

**Supplementary Figure S1:** Experimental set-up. Individuals were initially (Day 0) exposed to *Bsal* or maintained as negative controls; after 20 days, half of each group was exposed to 2,4-D (Day 20). Skin swabs were collected weekly to quantify *Bsal* load through qPCR. The trial ended 8 weeks after initial exposure to *Bsal* (Day 56). Morphometric measures were recorded at Day 0 and Day 56; buccal swabs to measure telomere length through qPCR were recorded on Day 56.


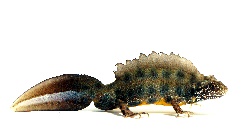

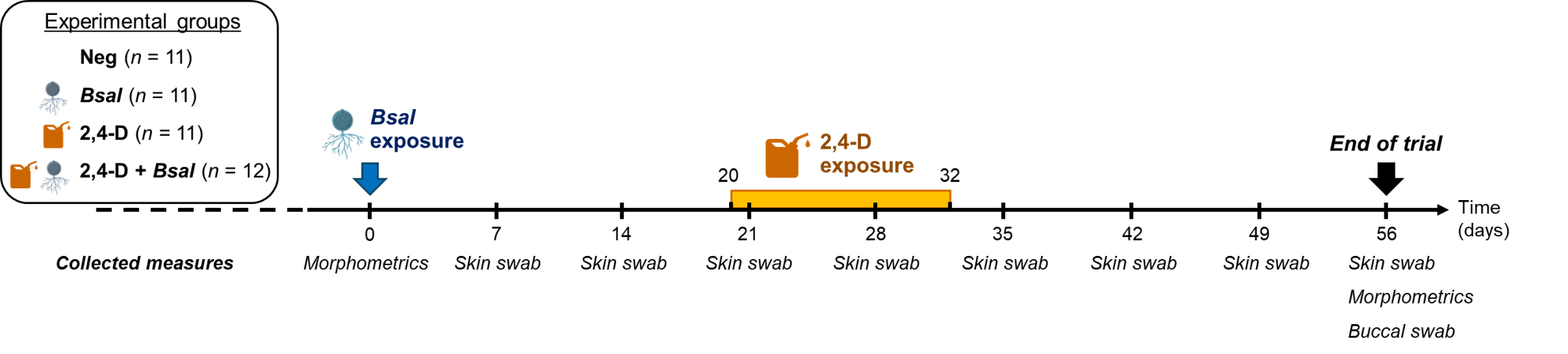


**Supplementary Figure S2.** Relation between initial body condition (Day 0) and total *Bsal* load (A), infection intensity (B) and disease severity (C) following exposure to *Bsal* (where each dot represents an individual, the black line shows the linear regression and the shaded area is the 95% confidence interval). Relation between 2,4-D treatment (2,4-D + *Bsal* vs. 2,4-D) and infection intensity (D) and disease severity (E) following exposure to *Bsal* (where each dot represents an individual, the black line is the median, the box shows the interquartile range (IQR), and the whiskers indicate the range of the data excluding outliers). The relation between 2,4-D treatment and total *Bsal* load is presented in Figure 2.


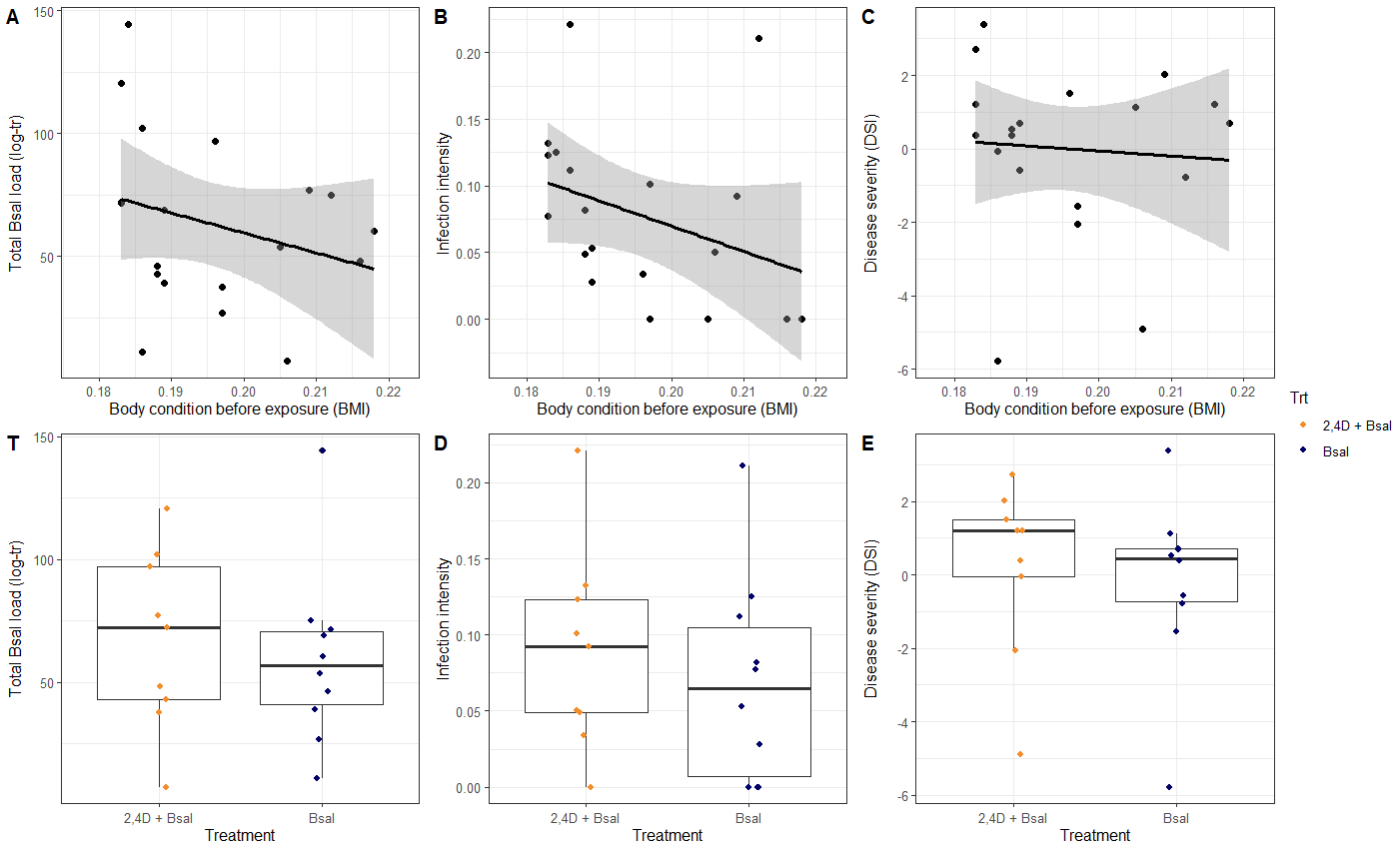


**Supplementary Figure S3.** Relation between final body condition and infection intensity (A). Relation between final telomere length and total *Bsal* load (B), infection intensity (C), and disease severity (D). Each dot represents an individual. The black line shows the linear regression and the shaded area is the 95% confidence interval.


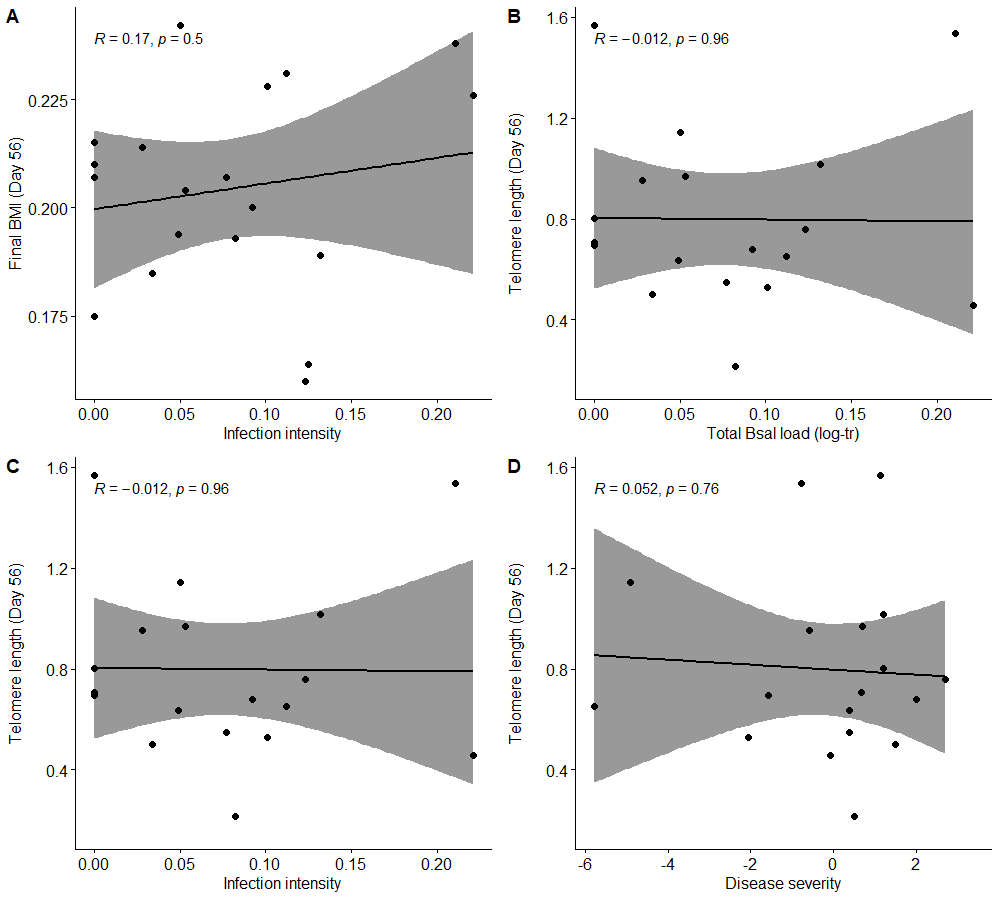


**Supplementary Figure S4.** Body condition change (delta BMI over the whole experiment) (A) and between variation in relative telomere length at the end of the experiment (B) across treatment groups*.* Each dot represents an individual. The black line is the median, the box shows the interquartile range (IQR), and the whiskers indicate the range of the data excluding outliers.


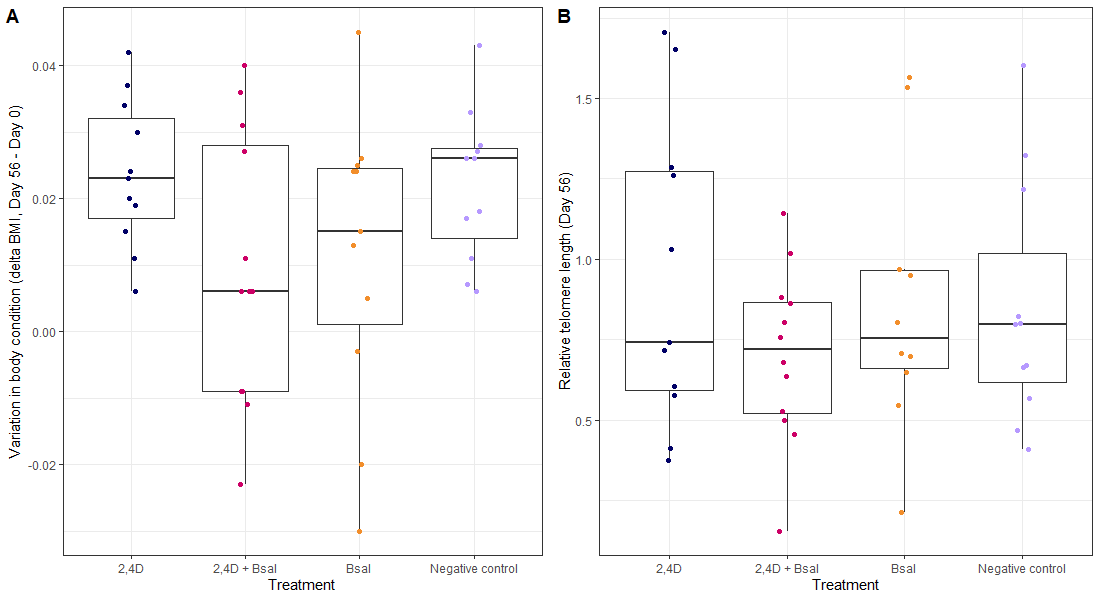


**Supplementary Material and Methods:** Build-up of the disease metrics.

For each individual, ***Infection Intensity*** was assessed as the slope to peak *Bsal* load within the first 4 weeks post-exposure (white area of the curves, early response to pathogen).


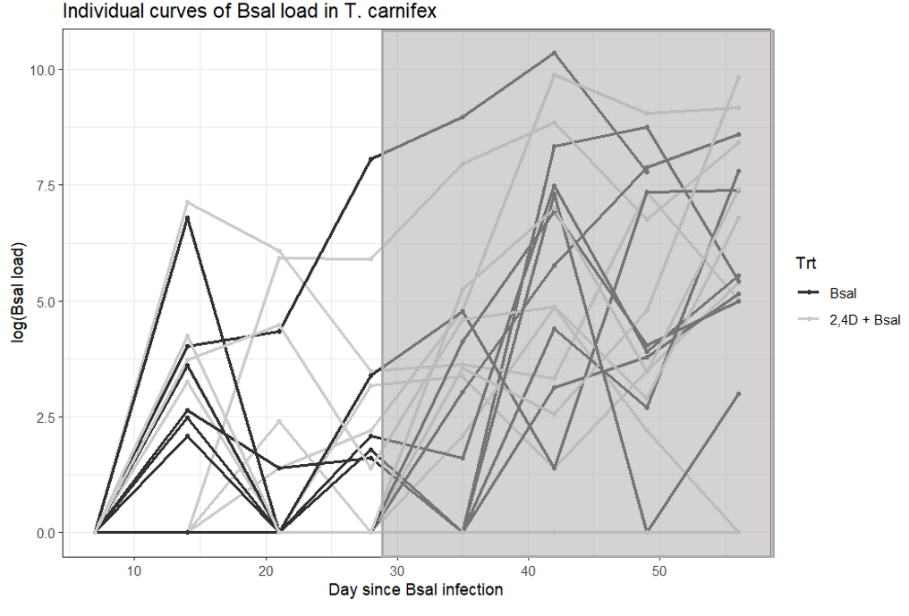


For each individual, the ***Disease Severity*** index was calculated as the sum of:

- its total *Bsal* load (pathogen burden), measured as the area under the curve of *Bsal* load drawn from weekly qPCRs (white + grey areas of the curves), standardized over the whole population;
- the latency to reach its peak *Bsal* load (infection kinetics), measured as the number of days between exposure to *Bsal* and day of maximal *Bsal* load for that individual, standardized over the whole population;
- its body-mass index (BMI) change (body condition), measured as the change in BMI between the last and first day of the experiment, standardized over the whole population;
- its survival indicator (time dead), measured as the time between the end of the experiment and the day of death, divided by the duration of the experiment. This indicator is comprised between [0;1] and null if the individual survived.

Note that in cases where an individual died, post-mortem *Bsal* load values were replaced with the last recoded *Bsal* load for that individual in order to compute the total pathogen burden.


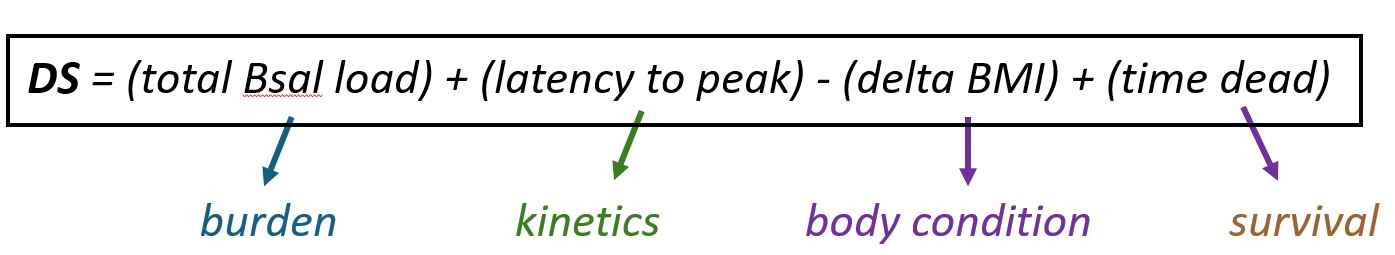


The R code used to generate these metrics is available in open source at Figshare repository.
